# Comparative Mask Protection against Inhaling Wildfire Smoke, Allergenic Bioaerosols, and Infectious Particles

**DOI:** 10.3390/ijerph192315555

**Published:** 2022-11-23

**Authors:** Jeff Wagner, Janet M. Macher, Wenhao Chen, Kazukiyo Kumagai

**Affiliations:** Environmental Health Laboratory Branch, California Department of Public Health, 850 Marina Bay Parkway, Richmond, CA 94804, USA

**Keywords:** aerosol models, exposure assessment, risk, COVID-19, wildfires

## Abstract

This work compares relative mask inhalation protection against a range of airborne particle sizes that the general public may encounter, including infectious particles, wildfire smoke and ash, and allergenic fungal and plant particles. Several mask types available to the public were modeled with respirable fraction deposition. Best-case collection efficiencies for cloth, surgical, and respirator masks were predicted to be lowest (0.3, 0.6, and 0.8, respectively) for particle types with dominant sub-micrometer modes (wildfire smoke and human-emitted bronchial particles). Conversely, all mask types were predicted to achieve good collection efficiency (up to ~1.0) for the largest-sized particle types, including pollen grains, some fungal spores, and wildfire ash. Polydisperse infectious particles were predicted to be captured by masks with efficiencies of 0.3–1.0 depending on the pathogen size distribution and the type of mask used. Viruses aerosolized orally are predicted to be captured efficiently by all mask types, while those aerosolized from bronchiolar or laryngeal-tracheal sites are captured with much lower efficiency by surgical and cloth masks. The predicted efficiencies changed very little when extrathoracic deposition was included (inhalable rather than respirable fraction) or when very large (100 µm) particles were neglected. Actual mask fit and usage will determine protection levels in practice, but the relative comparisons in this work can inform mask guidance for different inhalation hazards, including particles generated by yard work, wildfires, and infections.

## 1. Introduction

Masks have been recommended for the general public in recent years to reduce the risk of inhaling a range of potentially harmful airborne particles, such as wildfire smoke, influenza, or SARS-CoV-2 [1,2,3]. Masks also may be useful for those who suffer from seasonal allergies to pollen or fungi or who may be exposed to organic dusts, especially when performing increased-risk, non-workplace activities such as yard work, clearing brush, removing rodent droppings, or cleaning moldy materials [2,4,5,6]. Mask usage by the general public is a distinct activity from workplace mask usage, the latter of which is covered in the US by specific Federal and state regulations and guidance (e.g., [7,8,9]) and is not considered here.

Informing mask choices for the general public is challenging given the wide variety of mask types available. Although well-fitted respirator masks are the most protective choice, in practice, several factors may lead individuals to choose other mask types, including commercial availability, cost, individual fit, and comfort [2,10]. Of the available mask types, respirators (defined here broadly as including US NIOSH N95, European FFP2, Chinese KN95, etc.) are generally understood to be the most efficient, followed by surgical and cloth masks [3]. Substantial variation exists within each of these general classes, however. For example, substantial fit and performance discrepancies have been demonstrated for KN95 vs. N95 respirators, or different designs of cloth coverings [11,12,13,14].

Differences in mask performance may be attributed to differences in both face seal quality and material properties. Some degree of leakage is expected around the edges of surgical and cloth masks in particular; poor mask fit and improper usage can further increase mask bypass, leading to a significant decrease in effectiveness [15,16,17]. Currently only respirator masks are manufactured to efficiency and quality standards. Wide variation in performance, materials, and fit exist for surgical masks and cloth face coverings, though recent efforts have been made to standardize definitions for these types [6,18,19]. In addition, only respirator masks were expressly designed to provide inhalation protection for the exposed person rather than emission control.

Even neglecting leakage and defects, different mask materials possess different performance characteristics. Several studies have measured inhalation filtration efficiency for various respirator, surgical, and cloth media as a function of particle size [11,14,20,21,22]. These mask performance data depend substantially on the test aerosols and conditions, and the measured particle size distributions depend on the measurement instruments, conditions, and particle generation methods [23,24].

Despite these uncertainties, the use of generalizable aerosol science relationships can aid in assessing relative mask performance for different particle types. In the best case where mask fit is optimized, mask effectiveness for a given scenario depends upon the particle-size dependence of its efficiency curve and the size range of the particles of concern. Masks of all types are characterized by efficiency curves and efficiency minima at sizes determined by the superposition of various single-fiber filtration mechanisms (i.e., interception, inertial impaction, diffusion, gravitational settling, and electrostatic attraction) [25]. Most mask types are successful at filtering coarse particles (e.g., PM10–2.5 = particulate matter (PM) larger than 2.5 µm but smaller than 10 µm in diameter), but their ability to filter fine particles (PM2.5 = PM smaller than 2.5 µm in diameter) varies considerably. Wagner et al. [26] developed empirical mask models from literature data to represent relative filtration efficiencies for cloth, surgical, and respirator mask types.

Polydisperse particle size distributions can be characterized in terms of the median size and width of each of their constituent modes. Intact fungal spores (2–3 µm) and pollen grains (20–100 µm) are notable for their relative monodispersity [27]. Wildfire-emitted particles are somewhat dependent on fuel and burning conditions, but typically dominated by a mode smaller than 1 µm of fairly monodisperse, carbonaceous tar balls, and a second mode larger than 1 µm of inorganic ash particles [28,29,30].

Human-emitted particles have been revealed to be potentially much more polydisperse, due to a range of generation mechanisms and respiratory tract origins [23]. The physical size of a given pathogen determines the minimum infectious particle size, but the maximum infectious size and the infectious size distribution are not well understood. If the probability of human-emitted particles containing infectious virions or bacteria is assumed to be independent of particle size, then modeling the total emitted count distribution is appropriate. Various investigators have observed, however, that either smaller particles or larger particles are more infectious [31,32,33,34,35]. The true size distribution of infectious particles within the human-emitted size distribution may depend upon the infected organ location and illness progression [36]. Pöhlker et al. [23] provided a parameterization to estimate the size distribution emitted from each organ location. Nazaroff [37] used the human-emitted size distributions of Pöhlker et al. and the mask efficiencies of Wagner et al. to calculate fractional reductions in speaking emissions and particle inhalation from each respiratory mode.

This work expands upon the approach of Nazaroff to compare reductions in inhaled particle size distributions for a range of common aerosols that the general public may encounter. Size distribution measurements are compiled from the literature for wildfire smoke and ash, various allergenic aerosols, an infectious wind-borne fungus, and infectious bacterial and viral particles emitted by humans while breathing, speaking, or coughing. The reductions are calculated using empirical efficiency curves for respirator, surgical, and cloth masks. The human-generated infectious particle distributions are modeled as the sum of the infectious contributions from various organ sites, with minimum diameters set by the physical pathogen size. This information can be used to help determine how protective different mask types can be, assuming the best case of optimal mask fit, for a range of aerosol exposures.

## 2. Materials and Methods

Normalized, lognormal distribution modes [25] were used to model infectious microbial particles; wildfire smoke and ash; and allergenic, inflammatory, or irritant fungal and plant particles:

(1) $$N_{\mathrm{bz}}(d_{a})/N_{\mathrm{bztot}}=\frac{1}{\sqrt{2\pi }\times \ln GSD}\times e^{\frac{-{\left(\ln d_{a}-\ln CMD\right)}^{2}}{2\times {\left(\ln GSD\right)}^{2}}}$$

where *N_bz_*(*d_a_*) is the particle number concentration (particles cm^−3^) in the breathing zone for a given size bin, *N_bztot_* is the total particle concentration (particles cm^−3^) in the breathing zone, *d_a_* is the particle aerodynamic diameter (µm, assumed to equal the midpoint of the size bin), and CMD and GSD are the count median diameter and geometric standard deviation of the lognormal distribution, respectively. Equation (1) was discretized and evaluated at 12 particle size bins spanning *d_a_* = 0.02–420 μm. Note that *N_bz_*(*d_a_*) and *N_bztot_* are both generally divided by $\left[\ln\left(d_{a,\;h}\right)-\ln\left(d_{a,\;l}\right)\right]$, where *d_a,h_* and *d_a,l_* are the upper and lower boundaries of the size bin, but in this formulation these parameters cancel out.

Smoke and ash size distributions were modeled based on size data presented by Sparks and Wagner [28]. Allergenic bioaerosols were modeled using size distribution parameters from the literature [38,39]. The arthroconidia (Table 1) of soil-dwelling coccidioides represent a non-human-emitted, infectious fungal aerosol that is primarily transmitted naturally via wind and wildfires, and during active soil disturbance [40,41,42]. Because its infectious soil size distributions are not well-characterized, this work uses the known conidia distribution [43].

For human-emitted particles, polydisperse size distributions were modeled as the sum of multiple lognormal modes using the slightly different formulation and parameters of Pöhlker et al. [23]:

(2) $$N_{\mathrm{bz}}(d_{a})/N_{\mathrm{bztot}}=\sum_{i}A_{i}\times e^{\frac{-{\left(\ln d_{a}-\ln D_{i}\right)}^{2}}{\;{\left(Sigma_{i}\right)}^{2}}}$$

where *A_i_*, *D_i_*, and *Sigma_i_* are the lognormal parameters for individual modes representing specific organ origins (bronchiolar (B1 and B2), laryngeal-tracheal (LT), and oral (O1 and O2); see Table 1) that are summed for each particle size bin. *A_i_* in this case acts as a multiplier that represents the relative magnitudes of the different modes. By inspection of Equations (1) and (2), note that the lognormal parameters in the two formulations may be converted for a single-mode distribution (*A* = $\frac{1}{\sqrt{2\pi }\times \ln GSD}$, *D* = CMD, *Sigma* = $\sqrt{2}\times \ln GSD$).

Pöhlker et al. [23] incorporated a wider range of particle size measurements from the literature compared to those evaluated previously [21,26,47]. Both speaking and coughing exhibit LT emission modes not associated with breathing. Coughing is associated with a unique oral particle mode O1 at 11 µm, and both speaking and coughing are associated with another oral mode O2 at 100 µm. However, these large particle counts are insignificant compared to two strong, breathing-associated bronchiolar modes B1 and B2 at 0.07 µm and 0.3 µm, respectively, that Pöhlker et al. included in their speaking and cough models. The inclusion of these bronchiole fluid film burst modes is reasonable given that coughing and speaking are associated with breathing. However, the unique, transient exposures from speaking or coughing events may be important in some cases, independently of steadier bronchial contributions. In addition, the data supporting the presence of the smallest bronchial mode B1 are very sparse in Pöhlker et al.’s coughing data, and nearly non-existent in their speaking data. For these reasons, we present mask penetration results for speaking and coughing both with and without the smallest bronchial mode B1 included. In addition, the very large 100-µm particles predicted by Pöhlker et al. may be lost due to gravitational settling over a given distance, and thus may not be transported to an exposed person’s breathing zone. To simulate this effect, the calculations were repeated neglecting the largest 100 µm modes.

Infectious size distributions for influenza, tuberculosis, and SARS-CoV-2 were modeled by modifying the human-emitted particle distributions by two considerations. First, particle sizes smaller than the minimum pathogen physical size (viral [*d_a_* > 0.1 µm] [44,45] and bacterial [*d_a_* > 1 µm] [46]) were assumed to be non-infectious. Second, particles from different infection sites were compared to the default case (infectivity independent of particle size) by using isolated sub-components of the Pöhlker et al. model: (1) infectious pathogens aerosolized only from B1 and B2 sites (*d_a_* = 0.01–0.5 µm, modified by the minimum physical size of a given pathogen); (2) infectious pathogens aerosolized only from LT sites (*d_a_* = 0.5–2 µm); and (3) infectious pathogens aerosolized only from O1 and O2 sites (*d_a_* = 5–200 µm). Finally, limited measurements of infectious size distributions in the literature were compared to these modeled distributions. Table 1 presents the lognormal parameters for all modeled particle types, as well as the pathogen size that represents the minimum infectious particles size for a given type.

The collection efficiencies of the various mask types were determined using the empirical model of Wagner et al. [26]. This model assumes that masks obey general filtration laws dominated by diffusion, inertial impaction, and interception [25]:

(3) $$\begin{array}{cc} \eta_{mask}(d_{a}) & =1-\\ & \left[(1-\eta_{\mathrm{diffusion}})\times (1-\eta_{\mathrm{impaction}})\times (1-\eta_{\mathrm{interception}})\times (1-\eta_{\mathrm{interaction}})\right]\\ & =1-[(1-[A_{k}{d_{a}}^{-2/3}])\times (1-[B_{k}{d_{a}}^{2}])\times (1-[C_{k}d_{a}])\times (1-[D_{k}{d_{a}}^{0.17}])], \end{array}$$

where

*A_1_* = 0.040, *B_1_* = 0.017, *C_1_* = 0.158, *D_1_* = 0.266; *η_mask_* (*d_a_* > 6 mm) = 0.95, cloth mask

*A_2_* = 0.047, *B_2_* = 1.26, *C_2_* = 0, *D_2_* = 0.720; *η_mask_* (*d_a_* > 0.8 mm) = 0.95, surgical mask

*A_3_* = 0.063, *B_3_* = 0, *C_3_* = 0.743, *D_3_* = 1.04; *η_mask_* (*d_a_* > 0.7 mm) = 0.99, respirator

*A_4_* = 0, *B_4_* = 0, *C_4_* = 0, *D_4_* = 0, no mask

The data sets employed by Wagner et al. represent a range of mask fit conditions including human subjects, mannikins, and non-leaking custom apparatus [11,20,21,22], but all are likely a best case compared to the range of actual public practice because they were laboratory tested with due diligence and proper usage. In addition, the surgical masks in these data were likely of the multiple-layer, electrostatic type due to their relatively high observed measured efficiencies.

The overall collection efficiency *E*_tot_, summed over all 12 discretized sizes of a given modeled aerosol type, was obtained by multiplying the mask penetration $\left[1-\eta_{mask}\left(d_{a,\;j}\right)\right]$ by the inhalation fraction *F_inh_* [25] at each size *d_a_*, and then normalizing by the total concentration:

(4) $$\begin{array}{cc} E_{\;tot} & =1-\sum_{j}\frac{N_{inh}(d_{a,j})}{N_{bztot}}\;=1-\sum_{j}\frac{P(d_{a,j})\times N_{bz}(d_{a,j})}{N_{bztot}}\;\\ & =1-\sum_{j}\frac{\left[1-\eta_{mask}\left(d_{a,\;j}\right)\right]\times F_{inh}\left(d_{a,j}\right)\times N_{bz}\left(d_{a,j}\right){\;}^{}}{N_{bztot}}\;, \end{array}$$

where

$$\begin{array}{cc} F_{inh}(d_{a}) & =0.5\times \left[1+e^{-0.06\;d_{a}}\right]\times \left[1-e^{-e^{2.54-\left(0.681\;d_{a}\right)}}\right],\;\mathrm{respirable}\\ & =0.5\;\times \;(1+e^{-0.06\;d_{a}}),\;\mathrm{inhalable} \end{array}$$

*N_inh_*(*d_a_*)/*N_bztot_* is the normalized, inhaled particle concentration for a given size bin, and the overall penetration *P*(*d_a_*) is the size-dependent product of the mask penetration and inhalation fraction.

This work primarily modeled the respirable fraction (dominated by alveolar deposition in the deep lung), with limited tests of the inhalable fraction (nose and throat deposition) and the collection efficiency of the upper airways, nose, and mouth with no mask. All calculations were performed on a count basis. The inhalable fraction includes larger particles (>10 µm) that are generally extrathoracic, i.e., too large to be respirable [48] but still potentially important for allergens. Mask penetration and normalized inhaled particle concentrations were also plotted as a function of particle size.

## 3. Results

**Figure 1:**
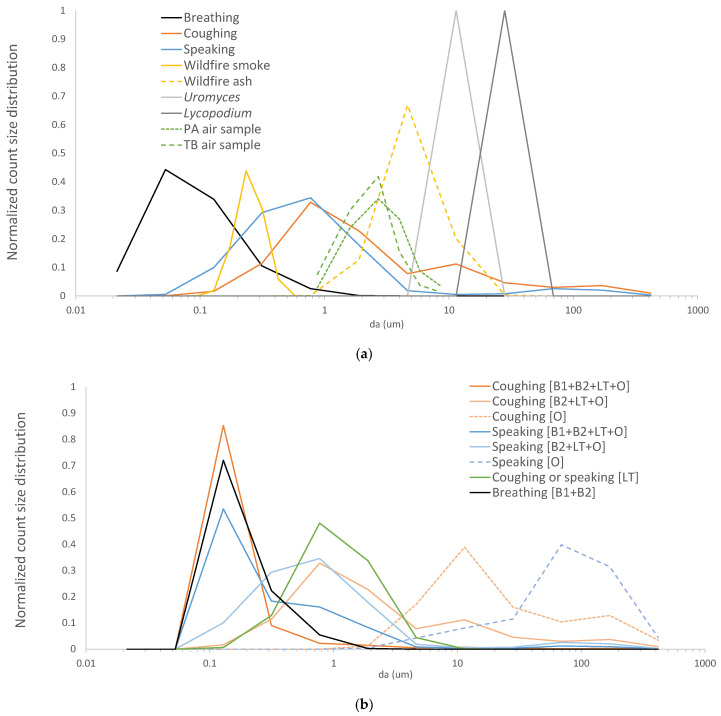
Modeled lognormal size distribution modes for: (**a**) allergenic bioaerosols (pollen grains and moss spores), human-emitted particles (coughs, speaking, and breathing), and wildfire-emitted particles (smoke and ash); and (**b**) infectious SARS-CoV-2 virion distributions generated at various organ locations. Also shown in (**a**) are data adapted from measured airborne *Mycobacterium tuberculosis* (TB) [49] and *Pseudomonas aeruginosa* (PA) [50]. All distributions were discretized and evaluated at 12 size bins spanning *d_a_* = 0.02–420 μm.

Figure 1 shows the wide range of particle sizes covered by lognormal distributions tested in this work (Table 1), including particles emitted by breathing, wildfire smoke, wildfire ash, fungal spores, particles emitted by speaking and coughing, and pollen grains. Also shown for comparison are data adapted from measured infectious particle distributions for *Mycobacterium tuberculosis* [49] and *Pseudomonas aeruginosa* [50].

Table 2 summarizes the mask efficiency predicted for all aerosol types, which for cloth, surgical, and respirator types were 0.3, 0.6, and 0.8 at their lowest, respectively. These poor performance levels corresponded to particle types with sub-micrometer modes coincident with minimum mask efficiency, such as wildfire smoke and coughing particles with the smallest B1 mode included. The larger minimum infectious size of mycobacteria compared to SARS-CoV-2 resulted in somewhat higher mask efficiencies for all human particle types. Influenza results were comparable to those of SARS-CoV-2 due to their similar primary sizes (Table 1) and are not presented in Table 2. All masks were predicted to achieve excellent efficiency (~1.0) for the largest-sized particle types, i.e., pollen grains and some fungal spores.

**Figure 2:**
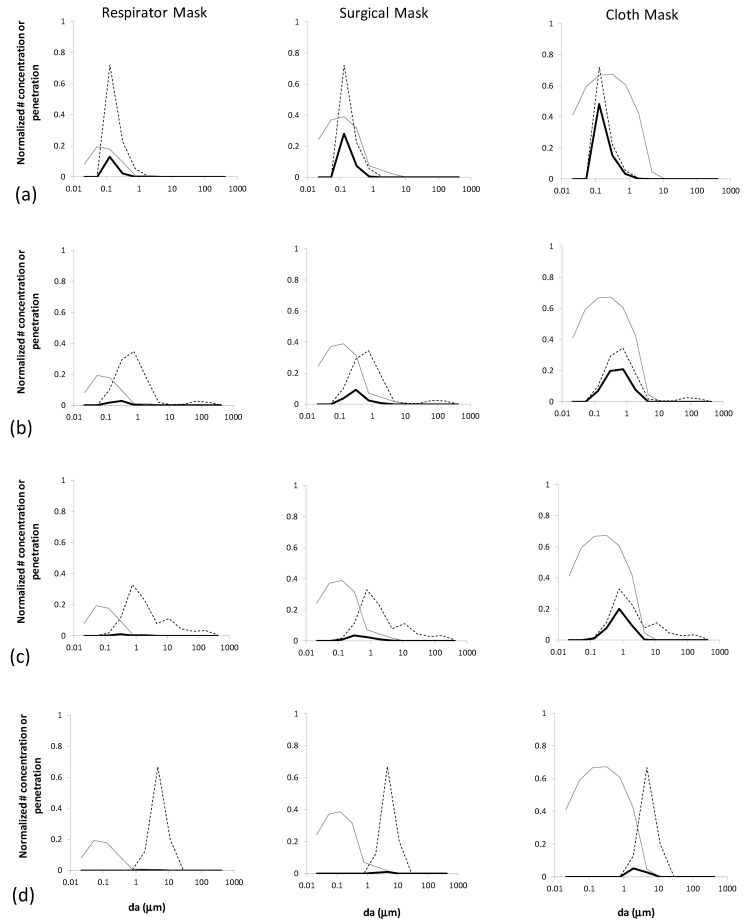
Comparison between mask penetration curves (*P* (*d_a_*), gray) and normalized size distributions in the breathing zone ([*N_bz_(d_a_)/N_bztot_*]/*dlogd_a_*), dashed) and lower lung ([*N_inh_(d_a_)/N_bztot_*]/*dlogd_a_*), solid) for three modeled mask types and: (**a**) breathing-emitted SARS-CoV-2; (**b**) speaking-emitted SARS-CoV-2; (**c**) coughing-emitted SARS-CoV-2; and (**d**) wildfire ash.

Normalized, inhaled count size distributions ([*N_inh_*(*d_a_*)/*N_bztot_*]/*dlogd_a_*) are compared in Figure 2 to pre-mask distributions in the breathing zone ([*N_bz_*(*d_a_*)/*N_bztot_*]/*dlogd_a_*) for four aerosol types and three mask types. It can be seen in Figure 2 that the overlap between the various mask penetration curves (P (*d_a_*)) and each aerosol type determines the size distributions that penetrate and reach the lungs.

Breathing-emitted particles can be seen to have substantial particle count penetration to the alveoli because they are fairly monodisperse and the majority of their particle sizes coincide with the maximal penetration for all mask types. In contrast, coughing- andspeaking-emitted particles have wider particle size distributions that overlap with only one side of the penetration curves, and thus a larger fraction that are collected by all mask types. Wildfire ash is dominated by larger particles, and thus has negligible lung penetration for all mask types except cloth masks, for which it is still greatly reduced compared to the breathing zone distribution.

**Figure 3:**
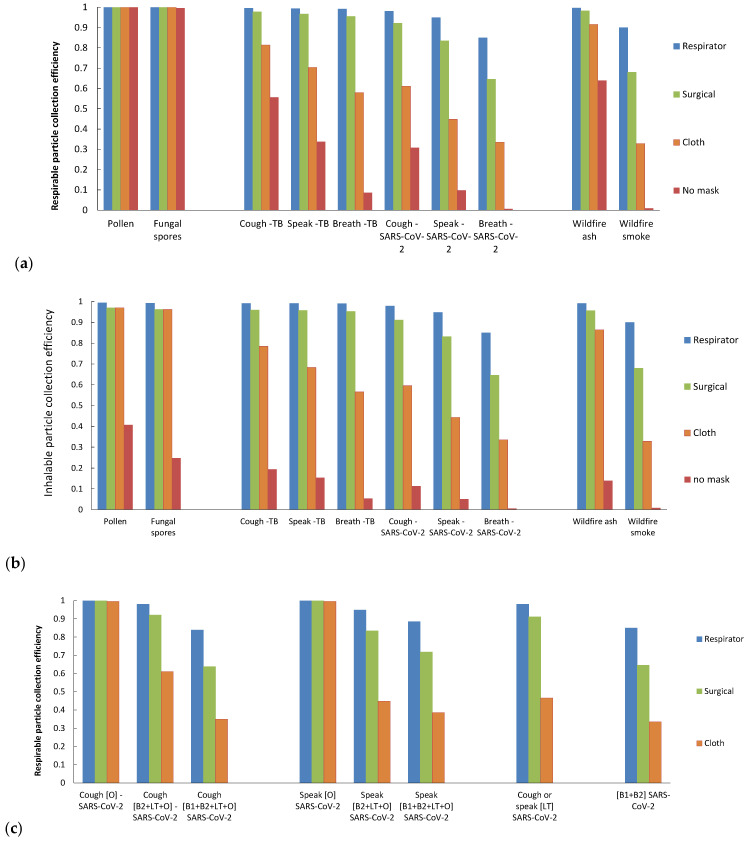
Predicted collection efficiency E_tot_ for (**a**) respirable particle deposition and respirator, surgical, cloth, or no masks for various aerosol types; (**b**) the same as (**a**) but for inhalable deposition, and (**c**) human-emitted particles with infectious SARS-CoV-2 generated at various combinations of organ locations for the three mask types.

Figure 3a shows the respirable particle collection efficiency for different mask types and a range of representative aerosol types. Although respirators were most efficient for all particle types, larger, allergenic bioaerosols (pollen grains and fungal and plant spores) were captured efficiently by all mask types. Surgical masks offered somewhat decreasing protection against infectious and wildfire particles, with worst performance for breathing and smoke particles. Cloth masks continued this downward trend even further. The lower protection for SARS-CoV-2 compared to *M. tuberculosis* was again due to the difference in modeled minimum sizes (0.1 and 1.0 µm, respectively). Although the inherent filtration of the upper airways with no mask offers little protection for the smallest particle types (human emitted and wildfire smoke), it effectively prevents the largest particle types (pollen and spores) from entering the lungs. The inhalable collection efficiencies in Figure 3b show very similar trends for all masks, except that no mask (mouth and nose filtration only) offers very little protection for any particle type. Figure 3c shows human-emitted particles with SARS-CoV-2 virions generated at various combinations of infected organ locations. Viruses aerosolized orally are predicted to be captured efficiently for all mask types, while those aerosolized from bronchiolar or laryngeal-tracheal sites are captured with lowest efficiency by surgical and cloth masks.

**Figure 4:**
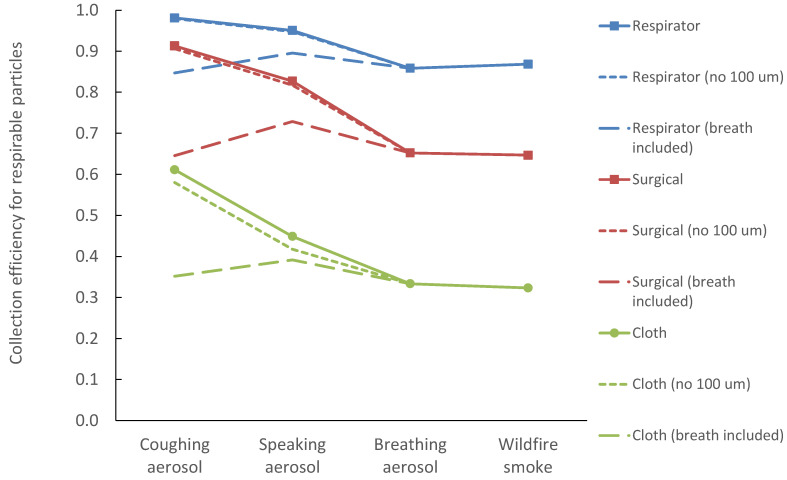
Collection efficiency E_tot_ for four aerosol types, compared to calculations with breathing-emitted particles included in coughing and speaking size distributions (coarse dashed line), and calculations that neglect the 100 µm particle modes of coughing and speaking (finely dashed line).

Including B1 bronchial modes in the coughing and speaking distributions made protection against all three types of human-emitted particles very similar (Figure 4). Conversely, neglecting the 100 µm particle modes in coughing and speaking particles yielded minimally reduced efficiencies. For respirable fraction calculations, these large particles represent a minor contribution to their total counts. While the largest particles are potentially important for mass-based efficiencies, all mask types are efficient at collecting these sizes (100 µm). A confounder here is the inability of these large particles to penetrate to the deep lungs. However, when the respirable calculations were repeated for the inhalable fraction (including the upper head airways), collection efficiencies were very similar.

**Figure 5:**
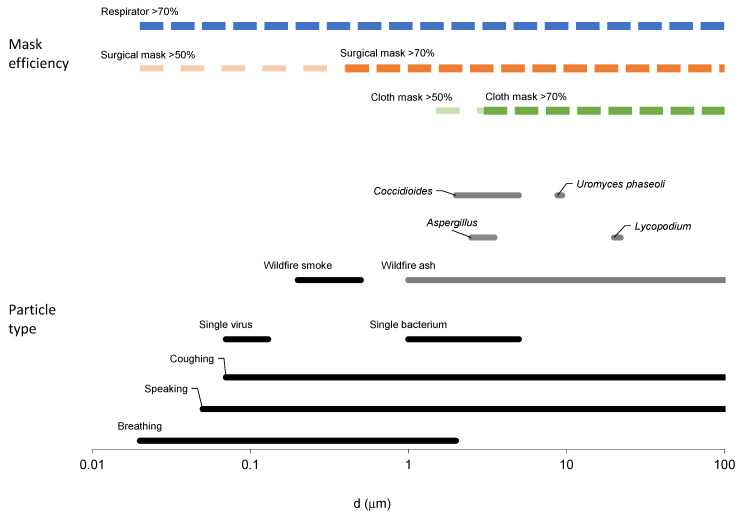
Size ranges for different particle types (**bottom of figure**) and the mask types predicted to have >50% and >70% efficiency at these sizes (**top of figure**). The coughing- and speaking-emitted particle size ranges are plotted independently of the bronchial mode B1 associated with breathing.

Figure 5 shows the ranges in which different mask types are predicted to have >50% and >70% efficiency compared to different particle-type size ranges.

## 4. Discussion

### 4.1. Impact of Particle Size Types on Mask Protection

This work employed various modeled aerosols spanning several decades in size to test a range of mask performance regimes. The modeled particles emitted by breathing, speaking, and coughing spanned five orders of magnitude (*d_a_* = 0.01–100 µm).

While respirator masks were predicted to be efficient for all particle types (efficiency = 0.8–1.0), surgical and cloth masks offered dramatically different protection against differently sized particles. In order of increasing surgical and cloth mask protection, wildfire smoke, ash, fungal spores, and pollen grains challenges resulted in efficiencies of 0.3–1.0. Similarly, polydisperse, infectious aerosols were predicted to be captured by surgical and cloth masks with efficiencies of 0.3–1.0 depending on the distribution of infectious agents within the emitted particle size range. Pathogens that were aerosolized orally were predicted to be captured efficiently for all mask types, while those aerosolized from bronchiolar or laryngeal-tracheal sites were captured with much lower efficiency by surgical and cloth masks. Singing is another mode of particle production that is similar to speaking, but with consistently higher emission rates [51]. Note that if breathing mode B1 is included in all human emission models, then speaking and coughing aerosols will be also captured with lower efficiency.

Figure 5 enables a relatively simple visual comparison of the different particle size ranges and the potential protection for different mask types. For example, surgical masks are predicted to protect with relatively high efficiency against the range that includes larger fungal spores and pollen grains, wildfire ash, and bacteria such as *M. tuberculosis*, and the largest wildfire smoke particles. Respirator masks are predicted to offer good protection against all particle sizes, whereas cloth masks are only highly protective against the largest allergens. Note that the smallest breathing-emitted particles are <0.1 µm and are thus not large enough to carry the virions considered in this paper.

### 4.2. Comparison to Experimental Data

Both measured airborne bacteria size distributions in Figure 1 [49,50] span approximately *d_a_* = 1–10 µm, comparable to the range reported by Asadi et al. [51] for speaking-generated particles. These measured infectious particle ranges are narrower than predicted for breathing-emitted particles, partially due to the minimum size constraint of the diameter of a single bacterium (on the order of 1 µm). The narrower measured ranges also may be due to limitations in instrumental measurement capabilities [23]. Recent breathing, speaking, and singing measurements near a single COVID-infected individual revealed SARS-CoV-2 RNA in slightly smaller particles (0.3 μm to >8 μm) [52], consistent with smaller virion diameters of approximately 0.1 μm.

The higher mask efficiencies predicted for larger allergens compared to sub-micrometer smoke and breathing-generated particles is consistent with numerous, particle size-dependent laboratory mask studies (e.g., [11,20,21,22]). However, more mask testing should be done under real-world conditions with specific particle types. Bergmann et al. reported pollen allergy reductions in adults wearing surgical masks that were as great as those wearing respirators [4], a result consistent with the large-particle predictions of the present work. Holm et al.’s summary of real-world studies of cloth, surgical, and respirator mask efficiencies (with ranges 0–50%, 20–70%, and 70–97%, respectively) [53] is broadly consistent with the mask models used here, though the particle types in each varied; one of these studies reported surgical mask efficiencies of 30% for particles < 2.5 µm and 70% for particles < 10 µm [54], consistent with the poorer protection for smaller particles predicted here.

### 4.3. Uncertainties and Limitations

Variations in head size, mask fit, and design features are likely to reduce these predicted efficiencies in practice via leakage. Respirator mask intervention studies have demonstrated reduced but still potentially useful protection (>70%) for children and other cases of sub-optimal fit [53]. Within the respirator mask class, different designs (e.g., head and neck straps for N95 vs. ear loops for KN95) may yield tradeoffs between comfort and performance, as demonstrated by laboratory tests [26,55]. Surgical masks without multiple electrostatic layers are likely to offer less protection than predicted here.

In addition, infectious, human-emitted particles may either shrink due to evaporation or agglomerate with ambient particles once airborne. Evaporation can shrink human-emitted particle sizes by 50–75% [37]. Because evaporation rates strongly depend on particle size, however, the smaller predicted modes in Table 1 likely already reflect their equilibrium sizes, while the larger particle modes may settle out before they shrink appreciably [23,37]. Agglomeration could result in slightly larger particles that would be collected at somewhat higher mask efficiencies than predicted here. However, combining with typical ambient size distributions (0.01–10 µm) likely would not increase the human-emitted sizes substantially.

Other factors potentially affecting inhalation mask efficiency but not considered in this work include particle liquid content as a function of temperature and humidity; inhalation rate dependence on exercise, speaking [56], individual physiology, or demographics; particle charge dependence; and degradation after multiple uses [57]. These issues should be explored further when refining models for mask guidance.

The distributions modeled in this work are count-based rather than volume-based. Previous authors have noted that the viral load of human-emitted infectious aerosols may depend on sputum volume [31,42]. If so, viral loads could be much higher for larger particles (volume~d^3^) with a corresponding shift in importance towards mask protection against larger particle sizes. This shift would favor cloth and surgical masks more than predicted in this paper and make their effectiveness closer to that of respirators. Field data instead show higher SARS- CoV-2 and influenza RNA counts in finer fractions [47,58], however, so the relative lack of large infectious particles may suggest that upper respiratory modes (O1 and O2) are not as important for SARS-CoV-2. Recent measurements of airborne *M. tuberculosis* yielded similar size distributions from coughing and speaking, also consistent with non-oral infectious contributions for both, though the measurements were not designed to capture the very large particles associated with coughing only [59].

Similarly, masks may offer greater protection than predicted here for wildfire smoke and allergenic/inflammatory particles if their health effects depend more on inhaled particle mass (e.g., allergen content) than count. Note, however, that lognormal, monodisperse size distributions do not shift as much to larger sizes when converted from count to mass as do polydisperse distributions [25].

This work was limited to investigating the relative protectiveness of masks against inhaling different types of particles. For human-emitted infectious particles specifically, masking in the general population also offers a second benefit of reducing source emissions from infected individuals [3,26,37,60]. Separate from the larger public health issues of mask guidance and mandates for the general public, this information may be useful for individuals who voluntarily wish to wear masks, such as those who suffer from seasonal allergies to pollen or fungi or who may be exposed to organic dusts, especially when performing increased-risk activities [4].

## 5. Conclusions

The use of general aerosol science relationships can aid in evaluating the relative suitability of masks for reducing inhalation of various particle types. In the best case where leakage is minimized, surgical and cloth masks are predicted to offer fair to good protection against pollen, spores, wildfire ash, and orally generated coughing and speaking infectious particles, but poor protection against breathing-emitted pathogens or wildfire smoke. When bronchial-generated particles are included in coughing and speaking size distributions, surgical and cloth masks have greatly decreased effectiveness for all particle types. As such, surgical and cloth masks represent an unavoidable performance compromise that should be evaluated against specific particle threats and the practical availability of respirator masks. Actual mask fit and usage will determine absolute protection levels in practice, but the relative differences in this work can inform better guidance for protecting against different inhalation hazards, including particles generated by yard work, wildfires, and infections.

## Figures and Tables

**Table 1:** Literature values, lognormal parameters for modeled particle type modes, and assumed minimum infectious particles sizes for different pathogens.

Particle Type	Reported by Source					Modeled in This Work	
	Range (µm)	*A* (#/cm^3^) *	*D* (µm) *	*Sigma* *	Source	CMD (µm) *	GSD *
Allergenic, inflammatory, or irritant and plant pathogenic particles							
*Lycopodium* species, *Ambrosia elatior*	20–22				[38]	21	1.1
*Uromyces phaseoli*	8.8–9.4				[38]	9.1	1.1
*Aspergillus* (*niger, flavus, fumigatus, japonicus, terreus*)	2.5–3.5				[39]	3	1.1
Wildfire smoke and ash							
Smoke	0.2–0.5				[26]	0.25	1.3
Ash	1–20				[26]	5	1.7
Human-emitted particles							
Coughing, bronchiolar mode 1 (B1)		262	0.07	0.9	[23]	Same as source	
Coughing, bronchiolar mode 2 (B2)		0.37	0.3	0.9	[23]	Same as source	
Coughing, laryngeal-tracheal (LT)		4.3	1	0.98	[23]	Same as source	
Coughing, oral mode 1 (O1)		1.4	11	0.95	[23]	Same as source	
Coughing, oral mode 2 (O2)		0.5	128	1	[23]	Same as source	
Speaking B1		9.8	0.07	0.9	[23]	Same as source	
Speaking B2		1.4	0.3	0.9	[23]	Same as source	
Speaking LT		1.7	1	0.9	[23]	Same as source	
Speaking O1		0.03	10	0.98	[23]	Same as source	
Speaking O2		0.17	96	0.97	[23]	Same as source	
Breathing B1		7.1	0.07	0.9	[23]	Same as source	
Breathing B2		1.1	0.3	0.9	[23]	Same as source	
Viral and microbial pathogens							
*Coccidioides* species	2–5				[43]	3	1.3
SARS-CoV-2	0.07–0.13				[44,45]	Min size = 0.1 µm **	
Influenza A, B, C	0.08–0.12				[46]	Min size = 0.1 µm **	
*Mycobacterium tuberculosis*	1–5				[46]	Min size = 1 µm **	

* *A*, *D*, and *Sigma* used in Equation (2); CMD and GSD used in Equation (1). ** Physical pathogen sizes used to set minimum infectious size in human-emitted particle distributions given above.

**Table 2:** Total respirable particle collection efficiencies predicted for different aerosol types. Human-emitted particles were modeled with three different minimum infectious sizes corresponding to SARS-CoV-2 (0.1 µm), *Mycobacterium tuberculosis* (TB) (1 µm), and no minimum size.

	Minimum Infectious Size	Collection Efficiency, E_tot_		
Aerosol		Respirator	Surgical Mask	Cloth Mask
*Lycopodium*		1.00	1.00	1.00
*Uromyces*		1.00	1.00	1.00
*Aspergillus*		1.00	0.98	0.86
*Coccidioides*		0.99	0.97	0.78
Wildfire ash		1.00	0.98	0.92
Wildfire smoke		0.90	0.68	0.33
Breathing (B1 + B2)	none	0.84	0.65	0.39
	SARS-CoV-2	0.85	0.65	0.34
	TB	0.99	0.95	0.58
Coughing (B1 + B2 + LT + O)	none	0.83	0.65	0.40
	SARS-CoV-2	0.84	0.64	0.35
	TB	1.00	0.98	0.81
Coughing (B2 + LT + O)	none	0.98	0.92	0.61
	SARS-CoV-2	0.98	0.92	0.61
	TB	1.00	0.98	0.81
Coughing (LT)	none	0.98	0.91	0.47
	SARS-CoV-2	0.98	0.91	0.47
	TB	0.99	0.96	0.62
Coughing (O)	none	1.00	1.00	0.99
	SARS-CoV-2	1.00	1.00	0.99
	TB	1.00	1.00	0.99
Speaking (B1 + B2 + LT + O)	none	0.86	0.69	0.41
	SARS-CoV-2	0.88	0.72	0.39
	TB	0.99	0.97	0.70
Speaking (B2 + LT + O)	none	0.95	0.83	0.45
	SARS-CoV-2	0.95	0.83	0.45
	TB	0.99	0.97	0.70
Speaking (LT)	none	0.98	0.91	0.47
	SARS-CoV-2	0.98	0.91	0.47
	TB	0.99	0.96	0.61
Speaking (O)	none	1.00	1.00	1.00
	SARS-CoV-2	1.00	1.00	1.00
	TB	1.00	1.00	1.00
	Min	0.83	0.64	0.33
	Max	1.00	1.00	1.00

## Data Availability

Data used to form conclusions are contained within the paper.
